# Fibroblast Growth Factor and Mineral Metabolism Parameters among Prevalent Kidney Transplant Patients

**DOI:** 10.1155/2012/490623

**Published:** 2012-07-02

**Authors:** Madhumathi Rao, Priyanka Jain, Temitope Ojo, Gautam Surya, Vaidyanathapuram Balakrishnan

**Affiliations:** Division of Nephrology, Tufts Medical Center, P.O. Box 391, 800 Washington Street, Boston, MA 02111, USA

## Abstract

*Background*. Chronic kidney disease (CKD) related mineral bone disorders persist after kidney transplantation, but little is known about the relationship between fibroblast growth factor-23 (FGF-23) and mineral metabolism in prevalent post-transplant patients. *Objectives*. To examine mineral metabolism parameters and their relationship to FGF-23 and parathyroid hormone (PTH) in prevalent kidney transplant patients. *Methods*. Cross-sectional study of 106 kidney transplant patients enrolled November 2005–October 2009 at Tufts Medical Center (TMC), Boston. *Results*. The prevalence of hypophosphatemia was 34%, hypercalcemia 3%, and elevated PTH levels 66%, at a median (25th–75th percentile) duration of 12.8 (7.5–30.9) months post-transplant. Males had significantly higher levels of PTH (*P* = 0.04) and lower levels of serum phosphate (*P* = 0.002). Serum PTH levels did not relate to eGFR, corrected calcium levels or serum phosphate. FGF-23 levels were above the reference limits in 7% of patients; higher levels were associated with higher serum phosphate and PTH levels after adjustment for transplant kidney function. *Conclusion*. FGF-23 is an important driver of mineral metabolism in prevalent transplant patients. Its modulatory role in mineral metabolism homeostasis may be heightened as feedback suppression of PTH is disturbed. Its role in long term cardiovascular and graft outcomes needs further study.

## 1. Introduction

Despite undergoing successful kidney transplantation, patients with chronic kidney disease (CKD) continue to have abnormalities in mineral metabolism. Persistent elevation of parathyroid hormone (PTH), sometimes autonomous, is well described, and hypophosphatemia owing to inappropriate urinary phosphate wasting with restoration of renal clearance is frequently seen in the early posttransplant period [1]. However, recent research also points to persistent posttransplant elevations of fibroblast growth factor-23 (FGF-23) as playing a major role in posttransplant hypophosphatemia and suppression of 1*α*-hydroxylase activity in the kidney [2, 3].

Although FGF-23 levels are reported to decline rapidly after transplant, there is limited information regarding its relationship to mineral metabolism in *prevalent* kidney transplant recipients. Kidney function is typically reduced in the transplant setting [4, 5], and the effects of transplant CKD on FGF-23 and mineral metabolism in the longer term are not well described. We report the results of a cross-sectional study where we examined abnormalities in mineral metabolism and their relationship to FGF-23 and PTH levels in prevalent transplant patients. As most studies have mainly addressed only the immediate changes following transplantation, this study represents an expansion in our understanding of the long-term effects of FGF-23 on mineral metabolism after transplantation.

## 2. Methods

### 2.1. Patients

 We enrolled 106 prevalent kidney transplant recipients between November 2005 and October 2009 from the outpatient nephrology clinic at Tufts Medical Center, Boston. Eligible patients were age 18 or older, who were between 6 months and 5 years posttransplant. Patients who had returned to dialysis, who had systemic illnesses such as an active malignancy, decompensated liver or respiratory disease, AIDS, acute intercurrent illnesses, pregnancy, or multiple organ transplants were excluded. Patients were not eligible for enrollment within 3 months of an acute rejection episode or acute kidney injury of any other cause. All participants provided written informed consent, and the study was approved by the hospital's institutional review board.

### 2.2. Study Design and Data Collection

 Using a cross-sectional study design, we collected demographic, clinical, and laboratory data and medication details from clinically stable kidney transplant patients at the time of enrollment by direct interview and supplemented relevant information from the subject's medical records. Laboratory test results related to mineral metabolism data consisted of measurements of serum calcium, phosphate, and albumin performed at the enrolment visit. Parathyroid hormone measurements were obtained within a 6-month window around the time of enrollment or where unavailable, measured in serum samples collected during the enrollment visit.

### 2.3. Definitions

*CKD *was defined as an eGFR of <60 mL/min/1.73 m^2^ and staged per NKF-KDOQI and KDIGO guidelines [5]. *eGFR estimation *was based on serum creatinine and the Modification of Diet in Renal Disease (MDRD) study equation [6]. An adjustment was made to account for the recalibration of serum creatinine assay performed by our laboratory during the study period. *Hypertension* was defined as BP >140/90 or the use of antihypertensive medication(s). The presence of *cardiovascular disease (CVD)* was indicated by the Index of Disease Severity (IDS) scores, a composite score for vascular disease-related variables [7].

Hypophosphatemia was defined as serum phosphate level <2.7 mg/dL. Elevated PTH levels were defined as a measurement >65 pg/mL [8]. Serum calcium was corrected for serum albumin levels [9], and hypercalcemia was defined as a serum calcium level >10.5 mg/dL.

### 2.4. Sample Collection and Assays

EDTA-anticoagulated blood samples were collected on ice at enrollment and immediately processed for separation of plasma and serum which were aliquoted and stored at −70°C until assay. Plasma levels of intact FGF-23 were measured using Human Intact FGF-23 ELISA kits (Immutopics, San Clemente, CA, USA). The reported assay parameters were sensitivity 1.0 pg/mL, intra-assay coefficient of variation (CV) 4.4% at 14.6 pg/mL and 2.6% at 148 pg/mL, and interassay CV, 6.1% at 15.6 pg/mL and 6.5% at 166 pg/mL. While reference ranges in the general population and clinical utility have not been established [10], previous studies using alternative assays have reported intact FGF-23 levels in healthy controls to range from 8.2 to 54.3 pg/mL and suggest a reference range of 10–50 pg/mL [11–13]. There has been a suggestion that the intact Immutopics assay may have inferior interassay variability; however, we assayed all samples in duplicate in a single sitting using assay kits from the same batch and therefore do not feel that this issue impacted our results.

### 2.5. Statistical Analysis

Statistical analysis was performed with the SPSS for Windows 14.0 (SPSS, Chicago, IL, USA). Data were expressed as means and standard deviations for continuous variables that were normally distributed and medians and interquartile ranges for nonnormally distributed data. Log transformation was carried out where appropriate (FGF-23, PTH, and phosphate levels). Categorical data were expressed as proportions. Mineral metabolism parameters were compared across demographic or clinical categories by the *t*-test or nonparametric tests as applicable. The geometric mean ± sem was computed where log transformation of variables had been performed. Correlations between continuous variables were analyzed using Spearman rank correlation. The determinants of plasma FGF-23 levels were examined using multivariable linear regression; similar analyses were used to compute the contribution of FGF-23 levels to the variability of serum phosphate. Assessment of model fit, residual analysis, and outlier detection were carried out for all regression models. All tests were two tailed, and *P* values <0.05 were considered significant. All confidence intervals were calculated at the 95% level.

## 3. Results

### 3.1. Clinical Characteristics

Table 1 shows the clinical and laboratory characteristics of the study population. The mean ± SD age of the population was 46.8 ± 11.2 years, 62% were male, and 12% were African-American; 55% of the subjects were posttransplant CKD stage 1T and 2T, 45% stage 3T and 4T. Roughly half the patients were between 1 and 5 years from their transplant date. Hypophosphatemia was present in 34% of the patients, hypercalcemia in 3%, and elevated PTH levels in 66%. The prevalence of CVD was 17%, and of documented bone disease was 21%, with radiological evidence of osteoporosis or osteopenia in 19% and avascular necrosis of bone in 2%. The immunosuppressive regimen predominantly consisted of tacrolimus and mycophenolate mofetil; about 45% used steroid-free regimens. Antihypertensive medications were used in 50% of the cohort. Approximately 50% of the study population was receiving treatment with either Calcitriol or Ergocalciferol, and 7% were taking phosphate binders. Males appeared to have significantly higher levels of serum PTH (geometric mean ± sem: 119 ± 1.1 pg/mL versus 83 ± 1.1 pg/mL in females; *P* = 0.04) and lower levels of serum phosphate than females (geometric mean ± sem: 2.8 ± 1.0 mg/dL versus 3.3 ± 1.0 mg/dL in females, *P* = 0.002). No significant differences in mineral metabolism parameters were seen by race, presence of diabetes, duration from transplant, presence of CVD, or bone disease.

### 3.2. Relationship between Mineral Metabolism Parameters and Transplant Kidney Function

eGFR was linearly inversely correlated to both serum calcium and phosphate, patients with higher eGFR having lower levels of both serum calcium and phosphate (Spearman's rank correlation −*r*, *P* value: serum calcium −0.27, 0.005; serum phosphate −0.20, 0.04). A higher eGFR was also associated with lower FGF-23 levels (Spearman's rank correlation −*r*, *P* value: −0.26, 0.006). However, neither eGFR nor calcium level showed any association with PTH levels, suggesting that the normal feedback mechanisms were disturbed in the posttransplant setting.

### 3.3. Plasma FGF-23 Levels and Its Determinants

 Median (25th–75th percentile) FGF-23 levels were 15.8 (12.2–22.5) pg/mL and had a right-skewed distribution. These levels were comparable to levels reported for healthy individuals in earlier studies and were above the reference limit of 50 pg/mL in 7% of patients. FGF-23 levels did not differ by age, sex, race, the presence of diabetes, or duration from transplant but were higher among patients with more advanced transplant CKD (median levels 14.5 pg/mL among patients with CKD stages 1 and 2 and 18.2 pg/mL among patients with CKD stages 3 and 4 (*P* = 0.02)).

**Figure 1:**
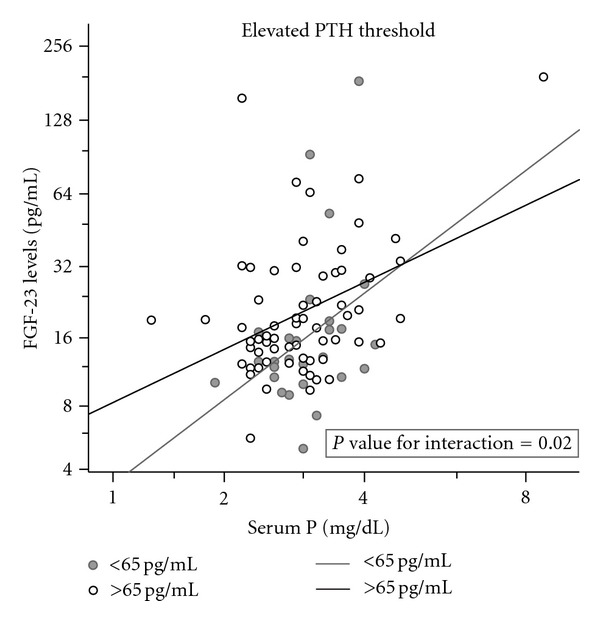
Scatterplot showing relationship of FGF-23 levels to serum P with and without elevated PTH levels. The slope of increase in FGF-23 with increasing P is less steep in the presence of elevated PTH levels (*P* value for interaction = 0.02; see Table 2 for coefficients).

Both higher levels of phosphate and higher PTH levels were associated with higher levels of FGF-23 (Spearman's rank correlation −*r*, *P* value: serum phosphate and FGF-23 = 0.29, 0.003; serum PTH and FGF-23 = 0.18, 0.06) and remained significant on adjustment for age, sex, race, transplant kidney function, and each other. The variation of FGF-23 levels with serum-phosphate levels completely explained its variability with transplant kidney function. The effect of phosphate levels on increasing FGF-23 levels appeared to be less marked among patients who had higher PTH and phosphate levels (*P* value for interaction 0.02, Figure 1). FGF-23 levels appeared to increase with duration from transplant, the highest levels being seen among patients beyond 2 years from transplant (geometric mean ± sem 19.9 ± 1.1 pg/mL beyond 2 years versus 14.1 ± 1.2 pg/mL less than 2 years from transplant, *P* = 0.02).  Table 2 shows the independent predictors for FGF-23 levels, the model explaining 22% of the variability of FGF-23 levels in this population.

### 3.4. Relative Contributions of FGF-23 and PTH in the Variability of Serum Phosphate

Serum-phosphate levels showed a weak inverse relationship to serum PTH levels (Spearman's rank correlation −*r*, *P* value: −0.21, 0.03) that disappeared after adjustment for gender, eGFR, and FGF-23 levels. Higher FGF-23 levels remained independently associated with higher serum phosphate levels, explaining 7% of its variability.  Table 3 shows the results of multivariable linear regression for phosphate levels, the model explaining roughly 23% of its variability, transplant kidney function contributing 7%, and gender 9%; respectively.

## 4. Discussion

In this study of 106 prevalent and clinically stable transplant patients with CKD stages 1T to 4T, between 6 months and five years from transplant, we found a high prevalence of abnormalities in mineral metabolism, with hypophosphatemia and PTH elevation. FGF-23 levels were not elevated in the majority of the patients, but higher levels were seen in the presence of higher serum phosphate and higher PTH levels. FGF-23 levels also increased with increasing duration from transplant, being highest in those patients >2 years from transplant. Although FGF-23 showed a significant inverse correlation with eGFR in an unadjusted analysis, this relationship was confounded by the relationship of serum phosphate with both eGFR and FGF-23 levels. PTH levels did not demonstrate either a relationship to eGFR or the usual inverse relationship to serum calcium levels, suggesting diminished responsiveness and either relative autonomy or substantial alterations in set point. FGF-23 levels appeared to be more important than PTH levels for phosphate homeostasis.

It is widely appreciated that FGF-23 level falls dramatically after transplant, but remains inappropriately elevated and continues to exert a phosphaturic effect in the early posttransplant period. Studies have demonstrated an inverse relationship between FGF-23 levels and serum phosphate [3, 11]. Evenepoel and colleagues suggested that this inappropriate hyperphosphatoninism subsides by the first year after transplantation [14]. Our results suggest that with increasing duration from transplantation, perhaps after an initial decrease, FGF-23 level continues to increase, and this change is more marked in patients with impaired transplant kidney function. Indeed, FGF-23 levels appear to increase in concert with serum phosphate, in contrast to the inverse relationship seen early after transplant, and this relationship is independent of transplant kidney function or PTH levels. In our results, PTH also appeared to act as an effect modifier, the presence of elevated levels decreasing the slope of increase in FGF-23 levels with increasing phosphate levels. However, as this study is cross-sectional, we are unable to address temporal relationships; it is neither possible to analyze if increasing levels of serum phosphate, albeit within the normal ranges, in prevalent transplant patients are the primary perturbation leading to FGF-23 elevation, nor if the modulation of this relationship by PTH levels is similar to the pathophysiology of CKD prior to transplantation.

 Disordered mineral metabolism, bone disease, and the consequences of vascular calcification persist after transplantation [15, 16]. Patients entering transplantation do so therefore with a significant CVD and risk factor burden with a high likelihood of disease progression [17–19]. Recently, FGF-23 has emerged as a powerful independent predictor of CVD outcomes and mortality in patients with CKD/ESRD [20–22]. Elevations in FGF-23 levels could potentially represent phosphate exposure and mediate its vascular consequences [23]. Furthermore, the adverse consequences of FGF-23 may also be affected through downstream inhibition of Vitamin D hydroxylation [24]. Calcitriol deficiency, observed in up to 45% of transplant patients [25], is associated with myocardial dysfunction, increased vascular calcification [26], as well as increased mortality [27–29]. A recent prospective study suggested that FGF-23 was a risk factor for kidney transplant loss and mortality, and the elevated risk was seen even with minor elevations of FGF-23 levels [30].

 Although the present study was cross-sectional at a single center, we obtained valuable insights into mineral metabolism relationships in a prevalent transplant population; moreover, the study population reflected a wide spectrum of transplant kidney function and posttransplant duration increasing the sensitivity to discover relationships and improve its generalizability. However, we acknowledge that our findings should be considered hypothesis generating and preliminary.

We also acknowledge that there is continuing debate regarding the best assay for the measurement of FGF-23 levels [31]. However, there is consensus that there is a good correlation between the different assays [10]. The use of the intact assay in this study was based on its specificity in the setting of decreased GFR where inactive C-terminal fragments could potentially accumulate. Interindividual variability in FGF-23 levels in CKD patients has been reported but in most instances appears to be less marked than the variability of other mineral metabolism markers such as serum phosphate or PTH, being in the range of about 10% [32, 33]. Despite these caveats, we found robust trends in the directions of relationships between FGF-23 levels and other mineral metabolism markers in this prevalent transplant population.

 In summary, FGF-23 appears to be an important driver of mineral metabolism after transplantation in prevalent transplant patients. Its relationship to serum phosphate differs compared to the early posttransplant period where its phosphaturic effect is more prominent. These results suggest that a phosphatemic stimulus to FGF-23 continues to exist after transplantation and increases in significance after the early posttransplant period especially in the context of impaired transplant kidney function. FGF-23 may play a more important role than PTH in the homeostasis of phosphate, given that the feedback regulation of PTH after transplant is relatively blunted. These changes may have major implications for long-term cardiovascular and graft loss outcomes in this population and highlight the need to study the modulatory role of FGF-23 in mineral metabolism homeostasis in the longer term after transplant.

## Figures and Tables

**Table 1:** Clinical and laboratory characteristics of patient cohort. Both numbers and percentages reported.

Age (years)	46.9 ± 11.2
Male	66 (62%)
Caucasian	93 (88%)
Diabetes (pre- or posttransplant)	16 (15%)
CKD	
Stage 1T & 2T	58 (55%)
Stage 3T & 4T	48 (45%)
Cardiovascular disease	18 (17%)
Bone disease	
Osteopenia/osteoporosis	20 (19%)
Avascular necrosis	2 (2%)
Multiple transplants	14 (13%)
Donor source	
Living donor	70 (66%)
Deceased donor	37 (34%)
Preemptive transplantation	33 (31%)
Prevalence of delayed graft function	16 (15%)
Received induction therapy	26 (29%)
Acute rejection episodes	15 (14%)
Immunosuppression	
Prednisone	58 (55%)
Tacrolimus	94 (89%)
Mycophenolate Mofetil	86 (81%)
Posttransplant duration (months)^∗^	12.8 (7.5–30.9)
eGFR (mL/min/m^2^)	62.9 ± 21.0
Serum calcium (mg/dL)	9.6 ± 0.5
Serum phosphate	3.1 ± 0.7
Serum PTH levels (pg/mL)^∗^	90 (58–172)
Hemoglobin (g/dL)	13.3 ± 1.8
Serum albumin (g/dL)	4.0 ± 0.3
Plasma FGF-23 (pg/mL)^∗^	16 (12–22)

^∗^median, 25th and 75th percentile, CKD: chronic kidney disease, eGFR: estimated glomerular filtration rate, PTH: parathyroid hormone, FGF-23: fibroblast growth factor-23.

**Table 2:** Multivariable linear regression for FGF-23 levels.

Variable	*β*	95% CI		*P* value
Serum phosphate (per log) (mg/dL)	4.56	1.46	7.67	0.004
PTH (per log) (pg/mL)	1.03	0.30	1.76	0.006
Serum phosphate (per log) ∗ PTH (per log)	−0.75	−1.36	−0.14	0.02
Duration from transplant >2 years (versus ≤2 years)	0.34	0.07	0.62	0.02

Model adjusted for age, sex, race, and eGFR. PTH: parathyroid hormone, CI: confidence interval.

**Table 3:** Multivariate linear regression for phosphate levels.

Variable	*β*	95% CI		*P* value
Sex (female)	0.14	0.05	0.22	0.002
eGFR (per 10 mL/min)	−0.02	−0.04	−0.005	0.02
PTH (per log) (pg/mL)	−0.04	−0.09	0.01	0.15
FGF-23 (per log) (pg/mL)	0.08	0.02	0.15	0.01

Model adjusted for age, sex, and race. eGFR: estimated glomerular filtration rate, PTH: parathyroid hormone, FGF-23: fibroblast growth factor-23.
